# Synthetic protein alignments by CCMgen quantify noise in residue-residue contact prediction

**DOI:** 10.1371/journal.pcbi.1006526

**Published:** 2018-11-05

**Authors:** Susann Vorberg, Stefan Seemayer, Johannes Söding

**Affiliations:** Quantitative and Computational Biology Group, Max-Planck Institute for Biophysical Chemistry, Göttingen, Germany; Linköping University, SWEDEN

## Abstract

Compensatory mutations between protein residues in physical contact can manifest themselves as statistical couplings between the corresponding columns in a multiple sequence alignment (MSA) of the protein family. Conversely, large coupling coefficients predict residue contacts. Methods for de-novo protein structure prediction based on this approach are becoming increasingly reliable. Their main limitation is the strong systematic and statistical noise in the estimation of coupling coefficients, which has so far limited their application to very large protein families. While most research has focused on improving predictions by adding external information, little progress has been made to improve the statistical procedure at the core, because our lack of understanding of the sources of noise poses a major obstacle. First, we show theoretically that the expectation value of the coupling score assuming no coupling is proportional to the product of the square roots of the column entropies, and we propose a simple entropy bias correction (EntC) that subtracts out this expectation value. Second, we show that the average product correction (APC) includes the correction of the entropy bias, partly explaining its success. Third, we have developed CCMgen, the first method for simulating protein evolution and generating realistic synthetic MSAs with pairwise statistical residue couplings. Fourth, to learn exact statistical models that reliably reproduce observed alignment statistics, we developed CCMpredPy, an implementation of the persistent contrastive divergence (PCD) method for exact inference. Fifth, we demonstrate how CCMgen and CCMpredPy can facilitate the development of contact prediction methods by analysing the systematic noise contributions from phylogeny and entropy. Using the entropy bias correction, we can disentangle both sources of noise and find that entropy contributes roughly twice as much noise as phylogeny.

## Author summary

Knowledge about the three-dimensional structure of proteins is key to understanding their function and role in biological processes and diseases. The experimental structure determination techniques, such as X-ray crystallography or electron cryo-microscopy, are labour intensive, time-consuming and expensive. Therefore, complementary computational methods to predict a protein’s structure have become indispensable. Over the last years, immense progress has been made in predicting protein structures from their amino acid sequence by utilizing highly accurate predictions of spatial contacts between amino acid residues as constraints in folding simulations. However, contact prediction methods require large numbers of homologous protein sequences in order to discriminate between signal and noise. A major obstacle preventing progress on the statistical methodology is our limited understanding of the different components of noise that are known to affect the predictions. We provide two tools, CCMpredPy and CCMgen, that can be used to learn highly accurate statistical models for contact prediction and to simulate protein evolution according to the statistical constraints between positions of residues as specified by these models, respectively. We showcase their usefulness by quantifying the relative contribution of noise arising from entropy and phylogeny on the predicted contacts, which will facilitate the improvement of the statistical methodology.

## Introduction

In the course of evolution, proteins are under selective pressure to maintain their function and correspondingly their structure. A possible mechanism to maintain structural integrity is the compensation of deleterious mutations between residue pairs in physical contact, known as compensatory mutations: Upon the mutation of one residue the contacting residue has an increased probability to mutate into a residue that will locally restabilize the protein structure, for instance by regaining a lost interaction between them. In multiple sequence alignments (MSAs) of related proteins, this effect leads to correlations between columns of residues in contact among most protein family members [1–4]. Many of these correlations are indirect, though, and arise through transitive chains of contacting residue pairs [5–8].

By applying statistical techniques that can distinguish mere correlation from direct statistical coupling of residue positions [5, 7, 9], many false positive predictions could be eliminated. The adoption of this class of statistical models, known as Markov random fields (MRFs), or Potts models in statistical physics, led to a breakthrough in de-novo (template-free) protein structure prediction: The predicted contacts proved sufficiently accurate to be used as spatial restraints to reliably predict protein 3D structures purely from sequence information [10–20].

The requirement for large MSAs for sufficiently precise predictions has limited the applicability of contact-assisted de-novo protein structure prediction, all the more because large protein families are more likely to contain at least one member whose structure has been solved and which can be used as a template for homology modelling. Therefore, most research has focused on making contact prediction reliable enough for medium-sized protein families [20–25].

The background noise effects arising in residue-residue contact prediction have been postulated to arise from three sources [5, 26–32]: random sampling noise due to the limited number of sequences, phylogenetic noise due to the evolutionary relatedness of sequences in the MSA, and entropic noise or rather bias, which biases high-entropy columns towards higher scores. Unfortunately, the relative contribution and properties of the three different sources of noise are difficult to study in real alignments, mainly because the true values of coupling parameters are not known. In addition, the stochastic noise, entropy-dependent noise and phylogenetic noise cannot be modified independently (for example by subsampling), as these noise sources are indirect, complex consequences of learning on only a limited number of sequences that are statistically dependent on each other according to their phylogenetic relationship.

Many correction schemes for removing noise from the matrix of predicted contact scores have been examined [27, 29, 30, 33–36], and the *average product correction* (*APC*) [26] came out as a clear winner and is used in almost all recent studies. However, it is widely acknowledged in the field that our limited understanding of what noise effects APC is correcting and why it is so effectively correcting them is severely impeding progress in developing better statistical methods to predict contacting residue pairs. We repeatedly made the experience that a promising extension to the standard MRF model that considerably improved the contact prediction performance *before* applying the APC was doomed to failure because it inexplicably yielded worse results than the baseline method *after* applying APC.

Based on theoretical considerations (Material and methods), we propose a simple entropy bias correction (EntC) that is computed solely from per-column entropies of the input MSA and corrects for entropy-dependent bias without affecting noise from phylogenetic effects. We find that the EntC eliminates nearly as much noise as the APC. The observation that both corrections can be expressed as a product of two factors depending only on each column separately explains partly the success of APC and suggests that it mainly corrects for entropy noise. Whereas the APC is applied as a post-correction to the matrix of predicted contact scores, the EntC can be applied directly on the statistical couplings of the MRF model, prior to computing a contact score and other post-processing treatments.

To systematically study the sources of noise limiting the accuracy of contact predictions from MSAs and to facilitate progress in the development of better contact prediction methods, we have developed CCMgen, a method for generating realistic synthetic protein sequence alignments whose residues obey the selection pressures described by a MRF with pairwise statistical couplings between residue positions.

For that purpose, CCMgen requires an exact statistical model that will reliably reproduce the empirical alignment statistics, such as single-site, pairwise or even higher-order amino acid frequencies, of the input MSA that was used to learn the MRF model in the first place. A typical strategy to obtain estimates of the MRF model parameters would involve maximizing the logarithm of the likelihood function over all sequences in the MSA. However, the normalization factor in the likelihood function requires to sum 20^*L*^ terms, where *L* is the protein length, and methods to optimize the full likelihood are very slow for realistic proteins [5, 37–41]. The most popular approximation is to maximize the pseudo-likelihood instead of the likelihood, as it can be shown that it converges to the same solution for large numbers of samples and it is fast to compute [42–44]. Even though pseudo-likelihood maximization gives results of the same quality of predicted residue-residue contacts as those using the full likelihood optimization, several studies unveiled that the pseudo-likelihood model is inaccurate and not able to accurately reproduce the empirical alignment statistics [37, 45].

We provide an implementation of an alternative precise inference technique, persistent contrastive divergence (PCD) [46] with our tool CCMpredPy. Compared to pseudo-likelihood maximization, PCD achieves identical precision for contact prediction while the inferred MRF model reproduces empirical marginals much more precisely. The increased quality of the models comes at the expense of longer run times, which are however still practical for even large proteins and alignments using a single desktop computer. High quality MRF models learned with PCD might prove beneficial beyond the purpose of contact prediction when problems require exact model statistics, e.g. when studying mutational effects or designing new protein features using the model energies.

Finally, we employ CCMgen in combination with MRF models that have been learned with the PCD algorithm and our entropy bias correction to quantify the relative effect sizes of phylogenetic and entropic bias on the precision of contact prediction. We find that the contribution of entropy noise in contact prediction is on average twice as big as that of phylogenetic noise.

## Results

### Persistent contrastive divergence allows accurate inference of MRFs

An exactly inferred MRF will reliably reproduce the empirical single-site and pairwise amino acid frequencies, *f*_*i*_(*a*) and *f*_*ij*_(*a*, *b*) for all positions *i*, *j* in the MSA and all amino acids *a*, *b* ∈ {1, …, 20} [7, 47]. Several studies demonstrated that pseudo-likelihood maximization, while being the method of choice for contact prediction, yields models that cannot accurately reproduce the empirical alignment statistics [37, 39, 45].

We developed a method that uses an inference technique called persistent contrastive divergence(PCD) [46] to learn MRF models that accurately reproduce the empirical alignment statistics. As in the study by Figliuzzi *et al.* [37], we computed for all Pfam MSAs in the PSICOV dataset the single-column and paired-column amino acid frequencies as well as covariances, $\text{cov}\left(\delta_{a,x_{i}},\delta_{b,x_{j}}\right)=f_{ij}\left(a,b\right)-f_{i}\left(a\right)f_{j}\left(b\right)$, where *δ*_*a*,*x*_ is the Kronecker symbol. We compared these statistics with those from sequences obtained by Markov chain Monte Carlo (MCMC) sampling from MRFs that were trained on the Pfam MSAs using either pseudo-likelihood maximization or PCD.

**Fig 1:**
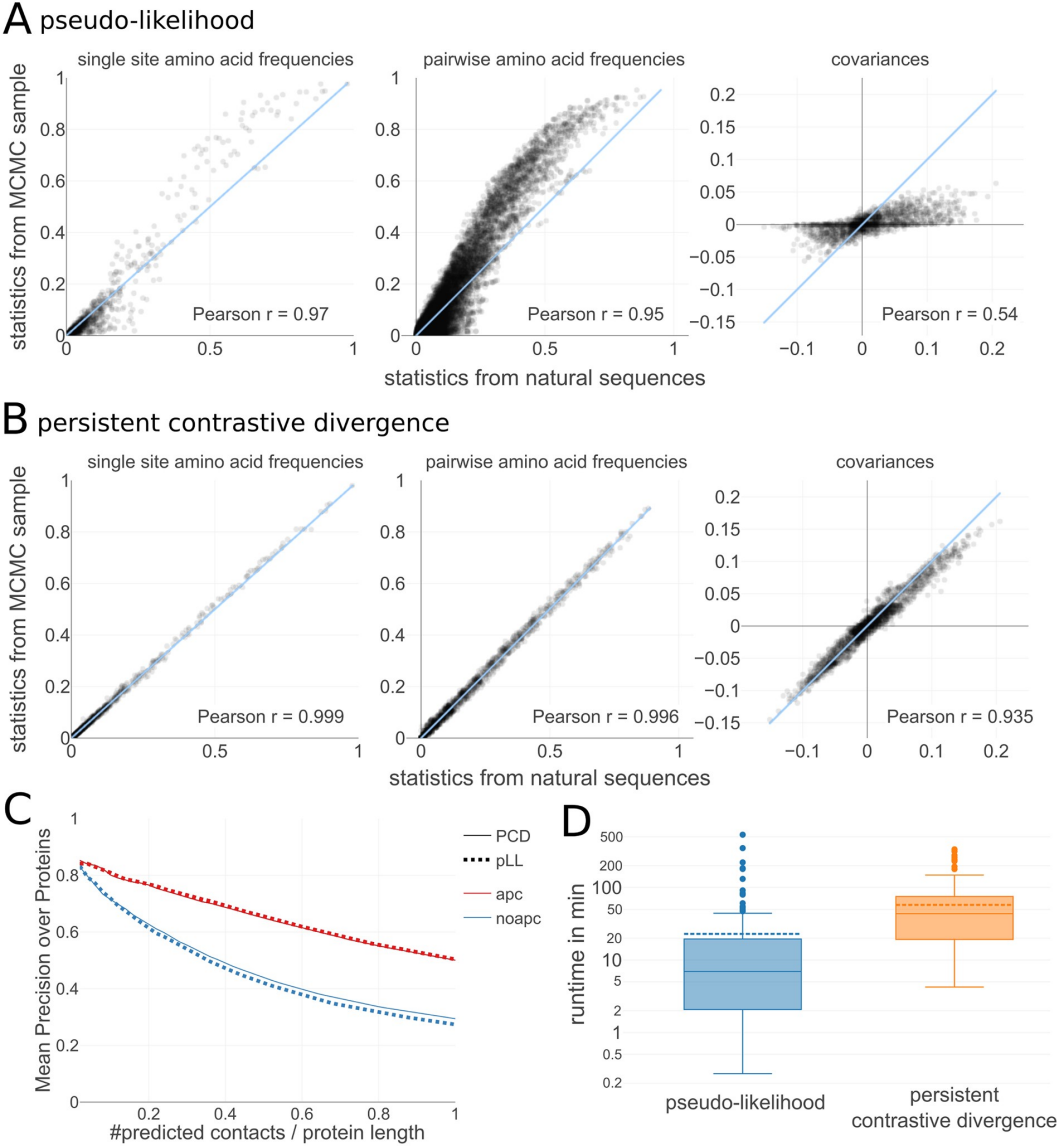
Persistent contrastive divergence permits the inference of high-quality models. **A** and **B** Comparing single amino acid frequencies (left), pairwise amino acid frequencies (center) and covariances (right) computed from natural sequences (Pfam alignment) and from Markov chain Monte Carlo (MCMC) samples generated from Markov random field (MRF) models trained with either pseudo-likelihood maximization (**A**) or persistent contrastive divergence (PCD) (**B**) for protein 1bkrA in the PSICOV dataset. **C** Mean precision of contacts, predicted as (APC corrected) *L*_2_ norm of pair couplings of a MRF trained with either pseudo-likelihood maximization or PCD. **D** Distribution of run times in minutes when learning MRF models with CCMpredPy (run on 4 cores). The median runtime in minutes with pseudo-likelihood is 7 minutes and with PCD is 43.5 minutes. Dashed line in boxplots represents the mean, solid line represents the median of the distribution. **C** and **D** are computed over the 150 proteins in the PSICOV dataset.

We find that the empirical single-site amino acid frequencies are well reproduced by both models. But whereas the empirical pairwise amino acid frequencies and covariances correlate strongly with the corresponding statistics computed from the PCD samples, the correlation is much weaker for samples obtained from pseudo-likelihood MRF models (Fig 1A and 1B and S1 Fig).

Furthermore, as in Figliuzzi *et al.* [37], we investigated how well the generated MCMC samples reproduce the alignment substructure of the original Pfam alignments with respect to the organisation of subfamilies in sequence space. We projected the protein sequences of the MCMC samples onto the first two principle components obtained from a principal component analysis (PCA) of the original Pfam MSA (for details see S2 Text). Again, we find that the alignment substructure described by the grouping of sequences that can be observed in the two-dimensional PCA space, is reproduced more reliably by MCMC samples generated from PCD models than from pseudo-likelihood models (S2b and S2d Fig).

It has been argued that for the purpose of predicting residue contacts an approximate model such as those obtained by maximizing the pseudo-likelihood for a MRF is sufficiently accurate to infer the correct topology of the interaction network of residues [45]. Fig 1C shows the mean precision of the predicted contacts from a PCD model and a pseudo-likelihood model versus the number of predictions per columns in the MSA. The precision for one MSA is the fraction of correctly predicted contacting pairs of positions (*i*, *j*) out of all predicted pairs. The correctly predicted pairs (*i*, *j*) are those for which the *C*_*β*_ − *C*_*β*_ distance in the reference protein structure of the Pfam MSA is below 8Å. Residue pairs that are separated by less than six positions along the protein sequence are not considered for the evaluation as they typically correspond to contacts within secondary structure elements and reflect local geometrical constraints. Indeed, predicted contacts from a PCD model achieve equal precision as predictions from a pseudo-likelihood model. S3 Fig shows further analysis, comparing the APC-corrected contact scores from pseudo-likelihood and PCD models.

However, more complex problems such as prediction of mutational effects or generating realistic samples of sequences, require exact model statistics. Several methods have been developed that exactly infer MRF models, such as bmDCA and ACE [5, 37–41], but they are computationally intensive which renders them impractical for real proteins. In comparison, our PCD-based CCMpredPy method is only about a magnitude slower than pseudo-likelihood maximization (Fig 1D).

### Correcting for entropy bias removes a major source of noise

A major obstacle for improving the statistical methods for residue-residue contact prediction is our lack of understanding of the sources of noise. The background noise effects have been postulated to arise from at least three sources, whose size and properties are difficult to quantify: phylogenetic, entropic and sampling noise.

**Fig 2:**
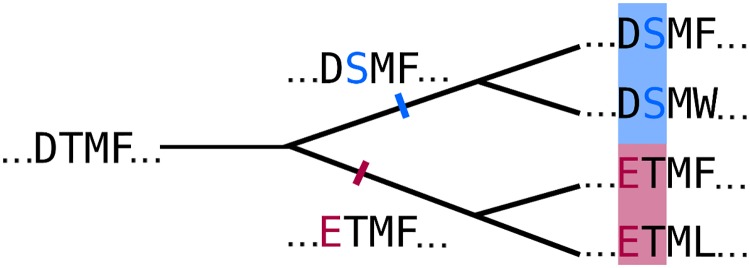
The phylogenetic dependence of closely related sequences can produce covariation signals. Here, two independent mutation events (highlighted in red and blue) in two branches of the tree result in a covariation signal for the two affected positions.

Phylogenetic noise originates from the violation of the assumption of independence of sequences in the MSA [48]. This assumption has been made by all methods that have been employed for contact prediction so far. To understand the origin of phylogenetic noise, consider the example in Fig 2. The MSA is composed of two subtrees whose last common ancestor sequences, DSMF and ETMF, had a mutation at the second and first position respectively. All descendants of the first ancestral sequence whose first two residues have not mutated in the meantime will have a DS at first and second position, while all descendants of the other ancestral sequence whose first two residues have not mutated yet will have a ET at those positions. Therefore, pairs DS and ET are more likely than would be expected from the frequencies of D and E in the first column and of S and T in the second column. The first and second position will therefore appear to be statistically coupled even though they are not.

Entropic bias describes the tight correlation of the expectation value of the contact score *c*_*ij*_ between columns *i* and *j* of a MSA under the assumption of no coupling between both columns with the product of the square roots of column entropies $s_{i}=-\sum_{a=1}^{20}f_{ia}\text{log}f_{ia}$:

$$\text{E}\left[c_{ij}\right]\underset{∼}{\propto }s_{i}^{\frac{1}{2}}\;s_{j}^{\frac{1}{2}}\;.$$ (1)

Put simply, higher column entropies lead to higher expected contact scores *c*_*ij*_ even if no coupling exists. To understand the origin of this bias, we need a bit of notation. From the MSA we compute coefficients *w*_*ij*_(*a*, *b*) that quantify the statistical coupling between residue *a* ∈ {1, …, 20} occurring in column *i* and residue *b* ∈ {1, …, 20} in column *j* of the same sequence (Materials and methods). A coefficient *w*_*ij*_(*a*, *b*) = 0.1 signifies that residue *a* in column *i* and residue *b* in column *j* in the same sequence is exp(0.1) times more likely to occur than what would be expected if the amino acids in both columns were independent of each other.

To predict contacts, we estimate the coupling coefficients *w*_*ij*_(*a*, *b*), for example by maximizing the pseudo-likelihood, and obtain estimates ${\tilde{w}}_{ij}\left(a,b\right)$, from which we can calculate a score to predict contacts. The commonly used contact score between columns *i* and *j* of a MSA is simply the norm of the 400-dimensional vector ${\tilde{w}}_{ij}$,

$$c_{ij}≔∥{\tilde{w}}_{ij}∥=(\sum_{a,b=1}^{20}{\tilde{w}}_{ij}(a,b{)}^{2}{)}^{1/2}.$$ (2)

It sums up the squared coupling coefficients over all possibly coupled amino acid pairs.

Let us assume that a MSA has no statistically coupled residue pairs, meaning that the true coupling coefficients are all zero. But the estimation of the coefficients results in errors, which contribute a systematic bias, as we will now see. The regularization of the MRF will ensure that the coupling coefficients *w*_*ij*_(*a*, *b*) for those amino acid pairs (*a*, *b*) without counts will be zero and will therefore not contribute to the overall contact score *c*_*ij*_ for this residue pair. For those pairs (*a*, *b*) with one or more counts, the *w*_*ij*_(*a*, *b*) will be distributed around zero but will rarely be exactly zero, just as *f*_*ij*_(*a*, *b*) is rarely exactly equal to *f*_*i*_(*a*) × *f*_*j*_(*b*). So each amino acid pair (*a*, *b*) that occurs at least in one sequence will make a contribution $\text{E}[{\tilde{w}}_{ij}{\left(a,b\right)}^{2}]$ to the sum in Eq 2. These contributions to *c*_*ij*_ stemming from noisy estimates *w*_*ij*_(*a*, *b*) create a bias that will increase with the number of pairs (*a*, *b*) of bins over which the *N* counts are distributed. Columns with high entropy tend to disperse the counts of amino acid pairs over more bins (*a*, *b*) than columns with low entropy. It is shown in Materials and Methods that the expectation value of this bias on *c*_*ij*_ can be approximated by a term proportional to product of the square roots of the entropies of the two columns.

The factorization of the EntC into two factors depending only on each column separately explains partly the success of APC and suggests that it mainly corrects for entropy noise (Materials and methods).

Sampling noise on the estimated coupling coefficients would remain, even if we correct for entropic bias and phylogenetic effects, because with a finite sample of sequences we cannot estimate fractions arbitrarily accurately. For example even if the sequences could be assumed to be independent of each other, the probability of an amino acid pair (*a*, *b*) that has been observed *n* ≪ *N* times out of *N* is only estimated to a relative accuracy of approximately $\sigma /\mu =\sqrt{n(1-n/N)}/n\approx 1/\sqrt{n}$, according to the standard deviation of the binomial distribution. More precisely, whereas the entropy bias describes the systematic offset of the contact score *c*_*ij*_ stemming from the non-zero expectation values $\text{E}[{\tilde{w}}_{ij}{\left(a,b\right)}^{2}]$, the sampling noise originates from the *variance* of the coefficients, $\text{var}[{\tilde{w}}_{ij}{\left(a,b\right)}^{2}]$, which is due to the finite number of measurements (sequences) *N* taken.

#### The APC and the EntC in action

**Fig 3:**
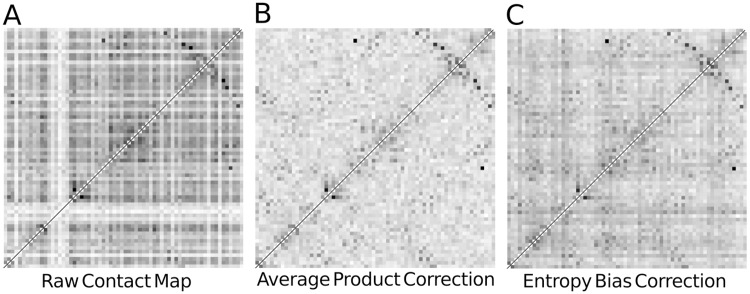
Entropy correction eliminates major source of noise. Raw and corrected contact score matrices for protein 1c9oA. The gray scale indicates the contact scores for each residue pair (*i*, *j*) for raw uncorrected scores computed from a Markov random field (MRF) model trained with persistent contrastive divergence (PCD) (**A**, Eq 2), average product corrected scores (**B**, Eq 17) and entropy corrected scores (**C**, Eq 18). The striping pattern in **A** arises from systematic score biases, which originate mainly from entropy.

Fig 3A shows the contact scores *c*_*ij*_ (see Eq 2) in grey scale computed from a MRF that has been trained from a typical example MSA. The striping patterns in horizontal and vertical directions reflect strong systematic row- and column-dependent score biases. Some positions seem to obtain generally higher scores than others. Without correction, ranking by these scores would severely overpredict contacts between positions with positive score bias and underpredict contacts between positions with negative bias.

Applying the APC eliminates the systematic effects leading to the striping patterns (Fig 3B). It thereby greatly improves the performance of all contact prediction scores for local pairwise measures such as mutual information as well as for global statistical coupling methods such as the MRF-based contact score referred to here [8, 26, 49–51].

To disentangle the entropic bias from the phylogenetic noise, we first propose an entropy-dependent correction, EntC, of the contact scores *c*_*ij*_ that depends solely on the per-column entropies *s*_*i*_ of the MSA from which the MRF was trained,

$$c_{ij}^{\text{EntC}}=c_{ij}-\alpha s_{i}^{\frac{1}{2}}\;s_{j}^{\frac{1}{2}}$$ (3)

with an analytically determined constant *α* that depends on all column entropies *s*_1_, …, *s*_*L*_. For a motivation of this score, see Materials and methods. Fig 3C shows how the entropy correction removes almost as much of the striping effects as APC.

**Fig 4:**
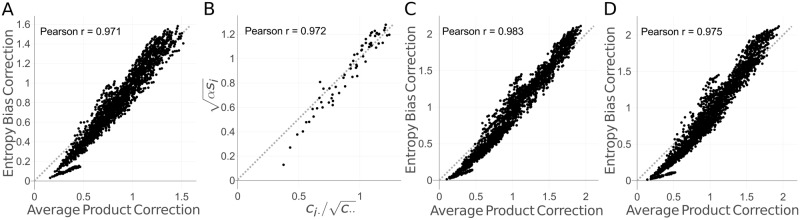
The average product correction (APC) and entropy bias correction (EntC) term correlate strongly. **A** For protein 1c9oA in the PSICOV data set the correction defined by the APC correlates well with the correction determined by our entropy bias correction strategy when learning a Markov random field (MRF) model with persistent contrastive divergence (PCD). **B** Also the factors appearing in the APC and EntC corrections, $c_{i\cdot }/\sqrt{c_{\cdot \cdot }}$ and $\sqrt{\alpha s_{i}}$., correlate well. **C,D** The Pearson correlation is similarly large when the MRF model is learnt from synthetic alignments generated with CCMgen using binary tree topologies (**C**) or using star tree topologies (**D**), both in the example of protein 1c9oA shown here and also across all 150 proteins in the Pfam dataset (S4 Fig).

Fig 4A reveals how strongly these two corrections correlate (see S4 Fig for the whole data set). Moreover, the correlation is high (Pearson correlation *ρ* = 0.972) also between the column-specific factors in the APC and EntC, that is, $c_{i\cdot }/\sqrt{c_{\cdot \cdot }}$ and $\sqrt{\alpha s_{i}}$ (compare Eqs 17 and 18). To analyze whether the correlation of APC and EntC is influenced by the phylogenetic dependence between the sequences, we generated synthetic MSAs with CCMgen using a binary and a star tree topology and trained an MRF on these two alignments (for details see next section). The correlation between the column-column APC and EntC correction terms remains as high as for the real Pfam MSA (Pearson correlations 0.983 (binary tree) and 0.975 (star tree) versus 0.971 (Pfam MSA)) (see Fig 4C and 4D). These results suggest that the APC predominantly corrects out entropy bias [26] rather than phylogenetic bias. Our theoretical analysis in Materials and Methods further supports this (see also the Discussion).

However, the relative contributions of entropic and phylogenetic noise limiting the precision of contact prediction are yet unclear. In the following we will use our tool CCMgen to distinguish between both sources of noise.

### Quantifying noise effects with CCMgen reveals entropy as dominating source of noise

**Fig 5:**
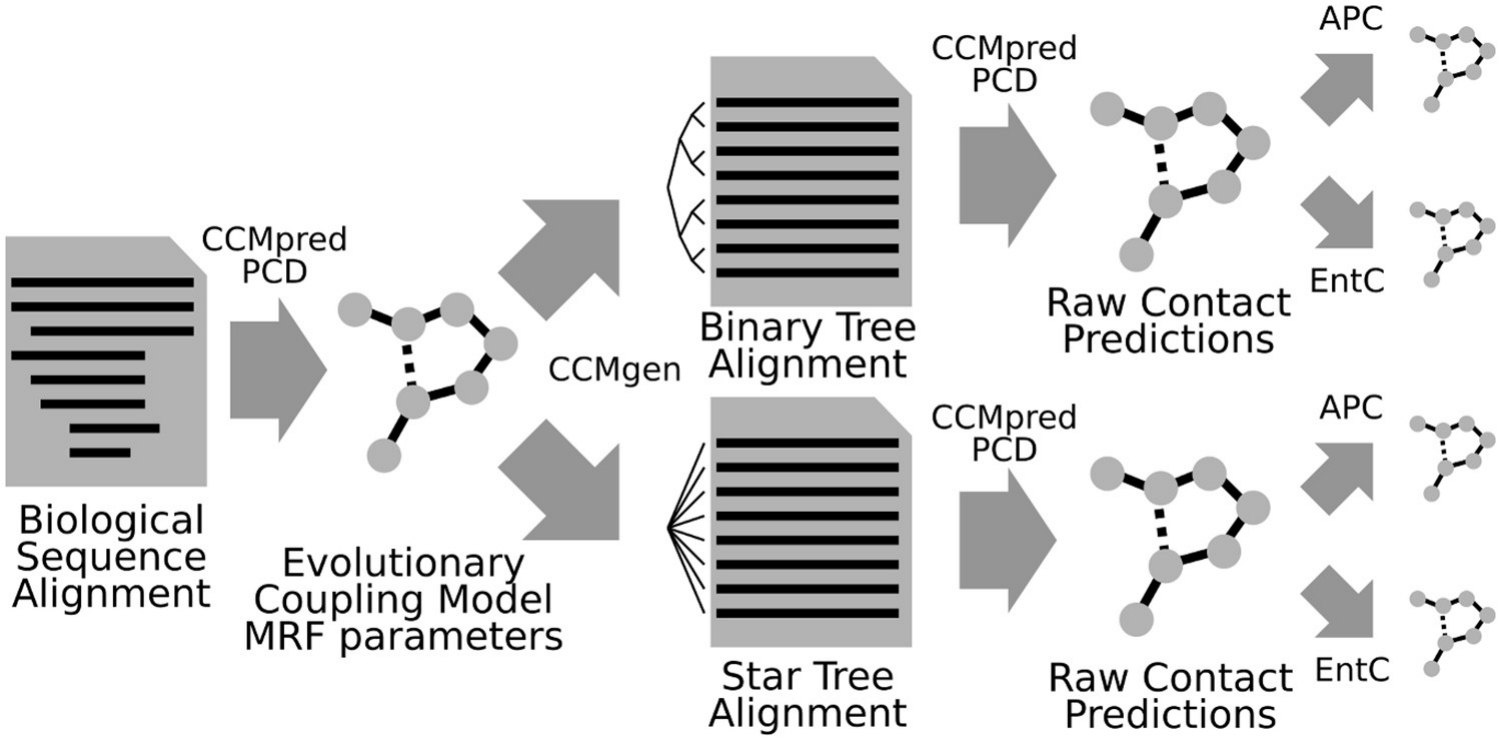
Workflow for quantifying noise effects. Artificial multiple sequence alignments (MSAs) are generated with CCMgen using a binary tree phylogeny for sequences with strong interdependencies and using a star tree phylogeny for nearly independently sampled sequences. Then, contacts are predicted and post-corrected using the average product correction (APC) and entropy bias correction (EntC)for both sets of alignments.

Our workflow to analyse the relative contributions of noise sources is described in Fig 5. First, we estimate the parameters of a second order MRF model with PCD using CCMpredPy for each of the 150 Pfam MSAs in the PSICOV data set. To obtain models with few but precise constraints, we set coupling parameters to zero for non-contacting residue pairs (*C*_*β*_ distance >12Å) during parameter learning.

**Fig 6:**
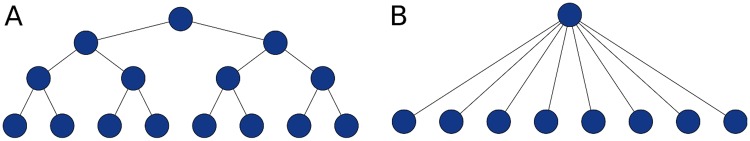
Idealized phylogenetic tree topologies available with CCMgen. CCMgen can generate multiple sequence alignments (MSAs) based on a Markov random field (MRF) model and a phylogenetic tree supplied either as Newick file or as one of the two shown, idealized topologies: **A** binary tree and **B** star-shaped tree.

In a second step, we use CCMgen with the learned model parameters to generate realistic synthetic MSAs of interdependent sequences with pairwise statistical couplings between some positions as they are observed in MSAs between residues in physical contact. CCMgen provides full control over the generation of the synthetic MSAs by allowing us to specify the evolutionary times and phylogeny along which the sequences are sampled. We sample two sets of synthetic MSAs: one set with a star tree topology and the other with a binary tree topology (Fig 6). Given sufficient evolutionary time, the phylogenetic dependencies between sequences drawn according to the star tree topology should be negligible, whereas sequences drawn along the binary tree are expected to contain stronger interdependencies.

Because the accuracy of predictions strongly depends on alignment depth and diversity [49, 52], we ensured that the synthetic alignments contain the same number of sequences and have similar diversities as the original Pfam alignments (for details see Material and methods). These provisions justify a direct comparison of the results for sampling sequences along the star and binary topologies.

Third, we run CCMpredPy on each of the synthetic MSAs and predict residue-residue contacts by ranking the pairs according to the descending raw contact scores (Eq 2), or by the APC-corrected contact scores (Eq 17) or by entropy corrected scores (Eq 18). Since we know the ground truth of which pairs are coupled from the MRF model used for generating the synthetic MSAs, we can use these alignments to investigate and quantify the effect of phylogenetic noise on the precision of residue-residue contact prediction.

**Fig 7:**
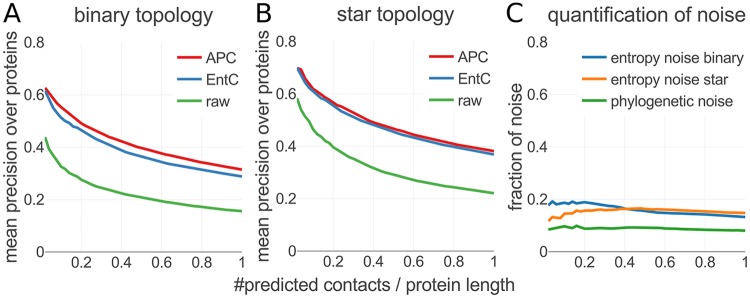
Effect of phylogenetic noise on contact prediction accuracy. MSAs generated with phylogenetic trees with star topologies do not contain phylogenetic noise. Therefore, the entropic bias is fully responsible for the difference in mean precision of predicted contacts between the uncorrected raw coupling scores (green) in (**B**) and the APC- or EntC-corrected scores. On MSAs generated with binary tree topologies, the precision of EntC-corrected scores drops due to phylogenetic noise (**A**). APC seems to correct out a small fraction of this noise, whereas EntC can only correct for entropy-related bias. (**C**) We can estimate the effect of phylogenetic noise as the drop in precision of the entropy-corrected scores (blue) from binary to star tree topology.

Fig 7A and 7B plot the mean precision of the predicted contacts from both types of synthetic MSAs versus the number of predictions per columns in the MSA. As expected, the mean precision drops as more predictions are considered and lower ranks are included.

Both APC and EntC correction have a huge effect in reducing noise and increasing the precision of predictions. Both corrections give very similar results for MSAs generated with star topology trees, which are not expected to show phylogenetic noise, while the APC performs slightly better than the EntC on MSAs with binary tree topologies. This suggests that the APC corrects out a small part of the phylogenetic noise. This would be plausible because this noise source affects some positions more than others (Fig 2) and would thereby also cause striping, which could be corrected by APC.

We estimate the strength of the phylogenetic noise as the drop in precision between the EntC-corrected precisions on MSAs with star topology and EntC-corrected precision on the MSAs with binary tree topology (Fig 7C). The strength of the entropy noise is shown in terms of the drop in precision between the EntC-corrected and uncorrected, raw contact scores, both for the star tree topology and for the binary tree topology. The contribution of entropy noise to the drop in precision is roughly two times larger than that of the phylogenetic noise.

## Discussion

### The average product correction explained

The success of the average product correction (APC) (Eq 17) is in part explained by three key insights: First, as we have seen, the entropy bias explains a large part of the noise in residue contact prediction. Second, as shown in Material and Methods, the EntC factorizes over columns, that is, it can be written as a product of two factors, each of which depends only on one column. Third, as we show now, the APC boils down to subtracting from the score *c*_*ij*_ an approximation to its expectation value under the null model of no couplings, if this expectation value factorizes into two terms, each of which depend only on one column,

$$\text{E}\left[c_{ij}\right]\approx u_{i}\;u_{j}\;.$$ (4)

Taken together, these three insights explain why the APC includes the EntC, which corrects for most of the bias.

To demonstrate the third insight, we approximate

$$c_{i•}=\sum_{j=1}^{L}c_{ij}\approx \sum_{j=1}^{L}E\left[c_{ij}\right]\approx \sum_{j=1}^{L}u_{i}u_{j}=u_{i}\langle u_{•}\rangle$$ (5)

$$c_{i••}=\sum_{i,j=1}^{L}c_{ij}\approx \sum_{i,j=1}^{L}E\left[c_{ij}\right]\approx \sum_{i,j=1}^{L}u_{i}u_{j}={\langle u_{•}\rangle }^{2},$$ (6)

because the sum over *L* terms averages out the fluctuations around the expectation value of each term. This approximation is probably the reason why the APC works better on *c*_*ij*_ than on $c_{ij}^{2}$ because for $c_{ij}^{2}$ the values in the sum are much more dispersed and dominated by one or a few terms, which renders the above approximation much less accurate. The APC correction is then

$$\frac{c_{i•}c_{•j}}{c_{••}}=\frac{u_{i}\langle u_{•}\rangle \langle u_{•}\rangle u_{j}}{{\langle u_{•}\rangle }^{2}}=u_{i}u_{j}=\text{E}[c_{ij}].$$ (7)

Hence the APC subtracts approximately the expectation value from *c*_*ij*_ if it factorizes over columns.

### EntC can overcome a major impediment to progress, the APC

As we have seen, the success of the average product correction (APC) (and other denoising techniques such as LRS [33]) depends on the specific form of the bias it can correct. The combination of pseudo-likelihood maximization, *L*_2_ regularization, and the definition of the contact score as the norm of the coupling vector $∥{\tilde{w}}_{ij}∥$ lead to a factorized form of the entropy, the leading cause of bias to correct for. It is plausible that changing the statistical model, its method of optimization, the regularization, or the contact score will usually result in the entropy bias to *not* factorize any more. For example, exchanging the *L*_2_ regularization by an *L*_1_ regularization destroys the factorization property. Therefore, even though the latter regularizer might work better, it can still perform worse after APC because APC does not correct its entropy bias well any more. A potentially very valuable result of this work is therefore the insight into what the APC actually corrects. If we can work out the expectation value of the contact score under the *L*_1_ regularization, for example, we could apply the appropriate entropy bias correction specifically for that model and regularization.

As another example, consider the following contact score, which uses amino acid pair-specific weights *β*_*ab*_ to upweight those pairs that are more predictive of contacts than others:

$$c_{ij}^{\prime }=\sum_{a,b=1}^{20}\beta_{ab}\;w_{ij}{\left(a,b\right)}^{2}\;.$$ (8)

The expectation value of this score does not factorize into separate terms for *i* and *j* any more and therefore the average product correction fails. Similarly, even neural networks would have a hard time to combine the coupling coefficients *w*_*ij*_(*a*, *b*)^2^ while learning to subtract the correct expectation value at the same time. This explains why it has been so difficult to improve on the popular combination of *L*_2_ regularization and contact score $c_{ij}=∥{\tilde{w}}_{ij}∥$ in combination with the APC.

But by subtracting the correct expectation value for each pair (*a*, *b*),

$$E\left[w_{ij}^{2}\left(a,b\right)\right]\approx \frac{N^{2}}{\lambda_{w}^{2}\left(N-1\right)}f_{ia}\left(1-f_{ia}\right)\;f_{jb}\left(1-f_{jb}\right)$$ (9)

we should be able to overcome this roadblock. For instance we can now define a score with weights *β*_*ab*_ whose expectation value under the null model is near 0, as it should,

$$s_{ij}=\sum_{a,b=1}^{20}\beta_{ab}(w_{ij}{\left(a,b\right)}^{2}-E\left[w_{ij}^{2}\left(a,b\right)\right])\;.$$ (10)

This equation allows for the correction of individual couplings *w*_*ij*_(*a*, *b*). It could therefore be used to train deep neural networks directly on the EntC-corrected coupling coefficients *w*_*ij*_(*a*, *b*), combining the advantages of entropy correction with learning directly from the full set of coupling coefficients [21, 24] instead of only from their EntC-corrected norms ∥*w*_*ij*_∥, as given in Eq 3.

### Persistent contrastive divergence facilitates inference of high quality models

Pseudo-likelihood maximization is the state-of-the-art inference technique for MRF models in contact prediction. Whereas the approximate nature of the model is sufficient for the correct ranking of residue pairs, the model is not exact in a way that it can reliably reproduce the empirical amino acid statistics of the original MSA. We implemented an alternative inference technique for MRFs, known as persistent contrastive divergence (PCD) which yields similar precision for predicted contacts but permits learning the fine statistics of the MRF model with higher precision. Even though other accurate model inference methods such as ACE [39] or bmDCA [37] can infer model parameters up to arbitrary precision, they are computationally intensive and their applicability is limited to small proteins. On the PSICOV dataset, our open source Python implementation of the PCD algorithm, CCMpredPy, was only about seven times slower than pseudo-likelihood maximization. (Its speed is proportional to the number of Markov chains and thereby depends on the required accuracy.) CCMpredPy might therefore be of use for large-scale studies that require exact models, such as investigating mutational effects or designing new protein features.

### CCMgen allows the generation of realistic synthetic alignments

We developed CCMgen, the first tool for generating realistic MSAs of protein sequences for a given phylogenetic tree whose residues follow the pairwise coupling constraints from a Markov random field model. CCMgen provides full control of parameters that determine the interdependencies between sequences through the specification of the phylogenetic topology and the evolutionary rate of the sampling process. It enables to distinguish different sources of noise observed in alignments and how they affect the performance of residue-residue contact predictions. We believe CCMgen will prove to be useful for improving and validating contact prediction methods.

In this study, we demonstrated how CCMgen can be applied to analyse the noise contributions from entropy and phylogeny. Given MRF models learnt on real MSAs, we generated synthetic MSAs with statistically coupled amino acid columns from two types of phylogenetic trees, one in which the sequences are maximally independent (star topology) and one in which the statistical dependences are much stronger (binary tree). By predicting contacts from the two types of synthetic alignments and correcting the predicted contacts either with the APC or with our proposed entropy bias correction, we were able to elucidate the effect of phylogenetic and entropic noise on contact prediction accuracy.

According to the quantification of noise effects, the most important goal for residue-residue contact prediction is an accurate treatment of entropic bias, as it accounts for roughly twice the amount of correctable noise and is especially important for correctly identifying the strongest evolutionary couplings. However, phylogenetic noise has an important contribution to the predictions and only a fraction of it is probably corrected by the popular average product correction (APC). This result shows that it might be very worthwhile to develop methods for contact prediction and for learning of MRFs that can explicitly take the statistical dependencies of sequences by common descent into account.

## Materials and methods

### Recap: MRFs model statistical couplings between columns in a MSA

To predict contacts between residues, a popular approach is to train a Markov random field (MRF) model describing the probability to observe a sequence **x** = (*x*_1_, …, *x*_*L*_) of length *L* with *x*_*i*_ ∈ {1, …, 20} representing the 20 amino acids,

$$p\left(x|v,w\right)=\frac{1}{Z}\text{exp}\;(\;\sum_{i=1}^{L}v_{i}\left(x_{i}\right)+\;\;\sum_{i<j}^{L}w_{ij}\left(x_{i},x_{j}\right)\;).$$ (11)

The couplings *w*_*ij*_(*a*, *b*) describe the preference to find amino acid *a* at position *i* and *b* at *j* in the same sequence in relation to the probability if these positions were independent, as parametrized by the single-column amino acid preferences *v*_*i*_(*a*). *Z* is the normalization constant, equal to the sum of the exp function in the numerator summed over all possible 20^*L*^ sequences.

To estimate the parameters *v*_*i*_(*a*) and *w*_*ij*_(*a*, *b*) of the MRF, the logarithm of the likelihood for all sequences in the MSA, equal to the sum over the log-likelihood of each sequence **x**_*n*_, could be maximized: $\sum_{n=1}^{N}\text{log}\;p\left(x_{n}|v,w\right)\to \text{max}$. A regularization term that pushes all parameters towards zero needs to be added to prevent overtraining, most commonly a *L*_2_ penalty,

$$R\left(w\right)=-\frac{1}{2}\lambda \sum_{i<j}^{L}\;\sum_{a,b=1}^{20}w_{ij}{\left(a,b\right)}^{2}.$$ (12)

But the huge number 20^*L*^ of terms in *Z* renders an exact solution infeasible for realistic protein lengths.

A number of approximations have been developed for this general class of problems. The approach that has consistently been found to work best for residue contact prediction is the pseudo-likelihood approximation, in which we replace the likelihood with the pseudo-likelihood and maximize the regularized log pseudo-likelihood [42–44],

$$\begin{array}{ll} PL\left(v,w\right) & =\prod_{n=1}^{N}\prod_{i:x_{ni}\neq 0}^{L}p(x_{ni}|x_{n,\backslash i},v,w)I\left(x_{ni}\neq 0\right)\\ PL_{\text{reg}}\left(v,w\right) & =\prod_{n=1}^{N}\prod_{i:x_{ni}\neq 0}^{L}\frac{1}{Z_{ni}}e^{v_{i}\left(x_{ni}\right)+\sum_{i<j}^{L}w_{ij}\left(x_{ni},x_{nj}\right)}+\exp\left(R\left(w\right)\right)\underset{v,w}{\to }\text{max}. \end{array}$$ (13)

Here, **x**_*n*,∖*i*_ denotes the vector obtained from **x**_*n*_ by removing the i’th component and $Z_{ni}=\sum_{c=1}^{20}\text{exp}(v_{i}\left(c\right)+\sum_{j:j\neq i}^{L}w_{ij}\left(c,x_{nj}\right))$ is a normalization constant, which can therefore be evaluated easily. The second product runs over all columns *i* for which *x*_*ni*_ is not a gap (represented by a 0).

Once the parameters **v**, **w** are estimated from a MSA, we can predict contacts for pairs of positions *i* and *j* using their statistical couplings. The most widely used score for residue contact prediction simply takes the *L*_2_ norm ∥**w**_*ij*_∥_2_ of the 20 × 20-dimensional vector **w**_*ij*_ with elements *w*_*ij*_(*a*, *b*) (Eq 2) [42, 43, 51, 53, 54]. In this study, we chose the regularization strength λ = 0.2(*L* − 1) [51].

### Sequence weighting and gap treatment

Sequences in a MSA do not represent independent draws from a probabilistic model. To reduce the effects of redundant sequences, we employ a popular sequence reweighting strategy that has been found to improve contact prediction performance. Every sequence *x*_*n*_ of length *L* with *n* ∈ {1, …, *N*} in an alignment with *N* sequences has an associated weight *ω*_*n*_ = 1/*m*_*n*_, where *m*_*n*_ represents the number of similar sequences:

$$m_{n}=\sum_{m=1}^{N}I\left(\text{Id}\left(x_{n},x_{m}\right)\geq 0.8\right)\;,$$ (14)

$$\text{Id}\left(x_{n},x_{m}\right)=\frac{1}{L}\sum_{i=1}^{L}I\left(x_{n}^{i}=x_{m}^{i}\right)\;.$$ (15)

An identity threshold of 0.8 has been used for all analyses. Amino acid counts and frequencies are computed with respect to the sequence weights. For example,

$$f_{i}\left(a\right)=\sum_{n=1}^{N}\omega_{n}I\left(x_{ni}=a\right)/\sum_{n=1}^{N}\omega_{n}$$ (16)

is the weighted fraction of sequences that have an amino acid *a* in column *i*.

We treat gaps as missing information and not as a 21st character. An example is Eq 13, where the second product runs over all MSA columns *i* except those having a gap in sequence *n*, *x*_*ni*_ = 0. This gap treatment leads to very minor changes both to the results and to the equations with respect to treating gaps as 21st character, e.g. the weighted number of sequences $\sum_{n=1}^{N}\omega_{n}$ gets replaced by $N_{i}=\sum_{n=1}^{N}\omega_{n}I\left(x_{ni}\neq 0\right)$ (the summed weight of sequences that do not contain a gap at positions *i* of the MSA), or by $N_{ij}=\sum_{n=1}^{N}\omega_{n}I\left(x_{ni}\neq 0,x_{nj}\neq 0\right)$. See, for example, Eqs 24 and 31 (for details see subsection 3.7.2 of PhD thesis of Susann Vorberg, available from soeding@mpibpc.mpg.de).

### Recap: Average product correction

The APC subtracts from each score *c*_*ij*_ = ∥**w**_*ij*_∥_2_ the product of the average score *c*_*i*•_ for row *i* times the average score *c*_*j*•_ for column *j* divided by the average score *c*_••_ over all cells [26]:

$$c_{ij}^{\text{APC}}=c_{ij}-\frac{c_{i•}c_{j•}}{c_{••}}.$$ (17)

The APC ensures that the average of the corrected coupling score over each column and over each row is 0. This can be verified by summing Eq 17 over all *i* or *j*. The assumption made is that, since each residue is only in contact with a small fraction of all residues, the mean coupling score over a column or row is dominated by the systematic score bias on all pairs in the column or row rather than by the coupling scores on a small fraction of contacting residues. APC can also be interpreted as an approximation to the first principal component of the raw contact matrix [33]. It therefore removes the highest variability in the raw contact matrix that is assumed to arise from background biases.

### Entropy correction

We define the following entropy bias correction (EntC), which depends solely on the per-column entropies of the MSA from which the MRF was trained:

$$c_{ij}^{\text{EC}}=c_{ij}-\alpha \;s_{i}^{\frac{1}{2}}\;s_{j}^{\frac{1}{2}}$$ (18)

where *α* is a coefficient determining the strength of the correction, and

$$s_{i}=-\sum_{a=1}^{20}f_{i}\left(a\right){\text{log}}_{2}f_{i}\left(a\right)$$ (19)

is the entropy of column *i*.

We determine *α* by analytically minimizing the sum of squares of the corrected off-diagonal coupling scores,

$$\sum_{i\neq j}^{L}(c_{ij}-\alpha \;s_{i}^{\frac{1}{2}}\;s_{j}^{\frac{1}{2}}{)}^{2}\to \underset{\alpha }{\text{min}}\;,$$ (20)

By setting the derivative to zero we obtain the optimal *α* value,

$$\alpha =\frac{\sum_{i\neq j}^{L}c_{ij}\;s_{i}^{\frac{1}{2}}\;s_{j}^{\frac{1}{2}}}{\sum_{i\neq j}^{L}s_{i}s_{j}}\;.$$ (21)

We also investigated other correction strategies using entropy statistics computed from the input MSA, such as the joint entropy for pairs of columns or different exponents in Eq 18. The resulting variations of the entropy correction performed comparably regarding the average correlation with APC as well as precision of contact predictions.

### Quantitative motivation of the entropy correction

We are given an MSA under the model that the sequences evolved under no pair couplings, that is, *w*_*ij*_(*a*, *b*) = 0 for all columns *i*, *j* and all amino acids *a*, *b*. The square of the coupling score for columns *i* and *j* is $c_{ij}^{2}=\sum_{a,b=1}^{20}{\tilde{w}}_{ij}{\left(a,b\right)}^{2}$, where ${\tilde{w}}_{ij}\left(a,b\right)$ are our estimates of the coupling coefficients learnt by maximizing the regularized pseudo-likelihood *PL*(**v**, **w**) in Eq 13. Our task is to calculate the expectation value of the coupling scores $c_{ij}=(\sum_{a,b=1}^{20}{\tilde{w}}_{ij}(a,b{)}^{2}{)}^{1/2}$. This expectation value under the null model of no couplings will be subtracted from the score to obtain the entropy-corrected score. For simplicity, we first assume that all sequences are independent draws from an MRF (with zero pair couplings).

From Eq 13 we derive the logarithm of the regularized pseudo-likelihood,

$$\begin{array}{cc} P\;L\;L_{\text{reg}}(v,w)= & \sum_{n=1}^{N}\sum_{i:x_{ni}\neq 0}^{L}\;(v_{i}\left(x_{ni}\right)+\;\;\sum_{j:j\neq i}^{L}\;\;w_{ij}\left(x_{ni},x_{nj}\right)-\text{log}Z_{ni}\left(v,w\right)\;)\\ & -\frac{\lambda }{2}\sum_{i\neq j}^{L}\sum_{a,b=1}^{20}\;w_{ij}{\left(a,b\right)}^{2}\;. \end{array}$$ (22)

At the local and global optimum, its partial derivatives with respect to the coupling coefficients must vanish:

$$\begin{array}{cc} \frac{\partial \;P\;L\;L_{\text{reg}}}{\partial w_{ij}\left(a,b\right)}= & \sum_{n=1}^{N}I\left(x_{ni}=a,x_{nj}=b\right)-\sum_{n:x_{ni}\neq 0}^{N}(\frac{1}{Z_{ni}\left(v,w\right)}\frac{\partial Z_{ni}\left(v,w\right)}{\partial w_{ij}\left(a,b\right)})\\ & -\lambda w_{ij}\left(a,b\right)=0\\ \frac{\partial \;P\;L\;L_{\text{reg}}}{\partial w_{ij}\left(a,b\right)}= & n_{ijab}-\sum_{n:x_{ni}\neq 0}^{N}\;p\left(x_{ni}=a|x_{n,\backslash i},v,w\right)\;I\left(x_{nj}=b\right)-\lambda w_{ij}\left(a,b\right)=0\;, \end{array}$$ (23)

where $n_{ijab}≔\sum_{n=1}^{N}I\left(x_{ni}=a,x_{nj}=b\right)$ counts how often *a* appears in column *i* at the same time as *b* in column *j*.

Under the hypothesis that none of the columns is coupled to any other and that the regularization λ is sufficiently strong, the estimated coupling coefficients ${\tilde{w}}_{ij}\left(a,b\right)$ will all be fairly small and scattered around zero. Therefore, the model probabilities $p(x_{ni}=a|x_{n,\backslash i},\tilde{v},\tilde{w})$ can be approximated by the empirical frequency $f_{ia}≔n_{ia}/N=\sum_{n=1}^{N}I\left(x_{ni}=a\right)/N$. Hence Eq 23 reduces to

$$\lambda {\tilde{w}}_{ij}\left(a,b\right)\approx n_{ijab}-\frac{N}{N_{i}}f_{ia}\;\sum_{n=1}^{N}I\left(x_{ni}\;\neq \;0,x_{nj}=b\right)\;.$$ (24)

Because under the null model gaps at position *i* occur approximately independently from *b* at *j*, $\sum_{n=1}^{N}I\left(x_{ni}\;\neq \;0,x_{nj}=b\right)\approx \left(1/N\right)\sum_{n=1}^{N}I\left(x_{ni}\;\neq \;0\right)\times \sum_{n=1}^{N}I\left(x_{nj}=b\right)=\left(N_{i}/N\right)\;Nf_{jb}$, we obtain

$$\lambda {\tilde{w}}_{ij}\left(a,b\right)\approx n_{ijab}-Nf_{ia}f_{jb}.$$ (25)

We now show that the counts *n*_*ijab*_ are distributed according to a *hypergeometric distribution*,

$$p\left(k=n_{ijab}|f_{ia},f_{jb},N\right)=\text{Hypergeom}\left(k=n_{ijab}|n,K,N\right).$$ (26)

with *k* = *n*_*ijab*_|, *n* = *Nf*_*ia*_, and *K* = *Nf*_*jb*_. Suppose you draw *n* objects (here: sequences) without replacement from a set of *N* objects, and *K* of these *N* objects have a certain feature (here: *x*_*nj*_ = *b*) while *N* − *K* don’t. Then the probability that *k* out of the *n* drawn objects have the feature is given by the hypergeometric distribution. In our case, the subset of objects = sequences that is drawn is the set of *n* = *Nf*_*ia*_ sequences that have an *a* in column *i*. The number *n*_*ijab*_ of these sequences that also have the feature *x*_*nj*_ = *b* is therefore distributed according to the hypergeomteric distribution.

The expectation value for a variable *k* = *n*_*ijab*_ is E[*n*_*ijab*_] = *nK*/*N* = *N* *f*_*ia*_ *f*_*jb*_. Therefore, the square of the coupling score *c*_*ij*_ can be expressed as

$$c_{ij}^{2}=\left|\right|w_{ij}{\left|\right|}_{2}^{2}=\sum_{a,b=1}^{20}{\tilde{w}}_{ij}{\left(a,b\right)}^{2}\approx \frac{1}{\lambda^{2}}\sum_{a,b=1}^{20}(n_{ijab}-\text{E}\left[n_{ijab}\right]{)}^{2}\;.$$ (27)

The expectation value of the numerator is (*n*_*ijab*_ − E[*n*_*ijab*_])^2^ = var[*n*_*ijab*_] which is $\frac{nK}{N}\frac{N-K}{N}\frac{N-n}{N-1}$, or, using our notation *n* = *Nf*_*ia*_, and *K* = *Nf*_*jb*_,

$$\begin{array}{cc} \text{E}[c_{ij}^{2}] & \approx \frac{1}{\lambda^{2}}\sum_{a,b=1}^{20}\frac{Nf_{ia}\left(1-f_{ia}\right)\;Nf_{jb}\left(1-f_{jb}\right)}{N-1}\;.\\ \text{E}[c_{ij}^{2}] & \approx \frac{N^{2}}{\lambda^{2}\left(N-1\right)}(\sum_{a=1}^{20}f_{ia}\left(1-f_{ia}\right))(\sum_{b=1}^{20}f_{jb}\left(1-f_{jb}\right))\;. \end{array}$$ (28)

Remarkably, the expectation value factorizes into a term depending only on *i* and one depending only on *j*. The factorization is in fact the reason why the APC (Eq 17) works so well, since the APC subtracts a product of two terms, $c_{i•}/c_{••}^{1/2}\times c_{j•}/c_{••}^{1/2}$, one depending only on *i* and the other only on *j*.

**Fig 8:**
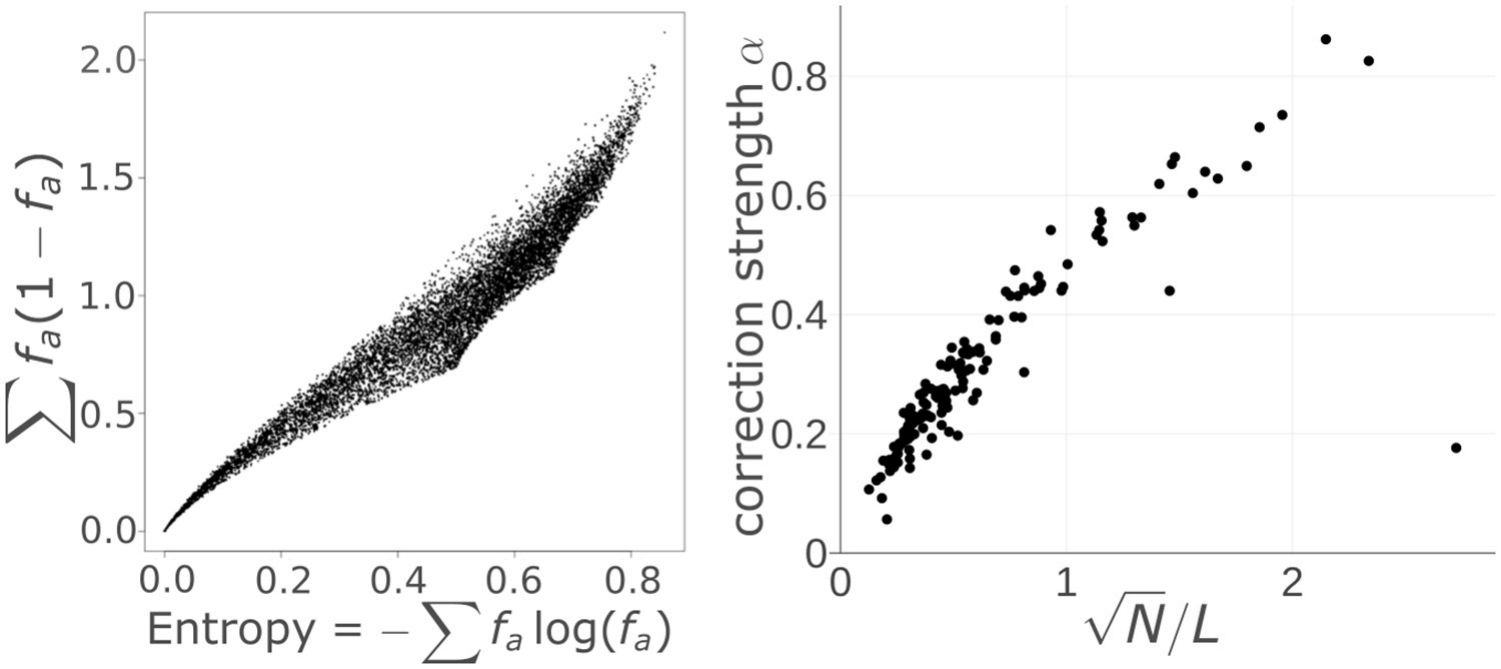
Entropy bias correction. (**A**) Each dot shows the entropy $-\sum_{a=1}^{20}f_{a}\text{log}f_{a}$ of a randomly sampled probability distribution over the 20 amino acids versus $\sum_{a=1}^{20}f_{a}\left(1-f_{a}\right)$ for the same distribution. (**B**) For each of the 150 Pfam MSA we plot the strength of the entropy bias correction *α* from Eq 21 versus $\sqrt{N}/L$, where *N* is the number of sequences in the MSA and *L* is the number of columns. Without sequence weighting, a linear dependence would be expected from Eq 30.

The factors in Eq 28 are highly correlated with the column entropies *s*_*i*_ (Fig 8A), so that we can write

$$\text{E}[c_{ij}^{2}]\underset{∼}{\propto }\frac{N^{2}}{\lambda^{2}\left(N-1\right)}s_{i}s_{j}\;.$$ (29)

Finally, the variance of *c*_*ij*_ is small in comparison to $\text{E}[c_{ij}^{2}]$ because usually many approximately independent terms (*n*_*ijab*_ − E[*n*_*ijab*_])^2^ contribute to $c_{ij}^{2}$ such that the fluctuations around the expectation value of each such term tend to average each other out. We can therefore approximate the entropic bias as

$$\text{E}[c_{ij}]=(\text{E}\left[c_{ij}^{2}\right]-\text{var}\left[c_{ij}\right]{)}^{1/2}\approx \text{E}{\left[c_{ij}^{2}\right]}^{1/2}\underset{∼}{\propto }\frac{N^{1/2}}{\lambda }s_{i}^{1/2}\;s_{j}^{1/2}\;.$$ (30)

Given that Eq 28 is a more accurate estimate for $\text{E}[c_{ij}^{2}]$ than Eq 30 is for E[*c*_*ij*_], we were expecting better results by predicting contacting residues (*i*, *j*) based on a ranking by $c_{ij}^{2}-\alpha u_{i}u_{j}$ with $u_{i}=\sum_{a=1}^{20}f_{ia}\left(1-f_{ia}\right)$ than when we ranked by the entropy bias corrected score $c_{ij}-\alpha s_{i}^{1/2}s_{j}^{1/2}$. To our surprise, the entropy bias correction worked slightly better. Investigation of this puzzling result is left for future work.

We used a regularization strength proportional to the number of residues *L* in the MSA, λ = 0.2*L*. Therefore, without sequence weighting, $\frac{N^{1/2}}{\lambda }$ should be proportional to the *α* parameter from Eq 21 that defines the optimum strength of the entropy bias. Indeed, Fig 8B shows a tight correlation of *α* with $\sqrt{N}/L$. We cannot expect the relationship to be strictly linear, because in our theoretical analysis we had assumed that sequences are independent and have a weight of 1, whereas in the example of Fig 8B the coupling coefficients ${\tilde{w}}_{ij}\left(a,b\right)$ where learned from Pfam MSAs using sequence weighting.

### Learning MRFs with persistent contrastive divergence

While the log likelihood function cannot be efficiently computed because of the exponential complexity of the normalization constant *Z*, it is possible to approximate its gradient with an approach called contrastive divergence [55]. The gradient of the log likelihood with respect to the couplings *w*_*ij*_(*a*, *b*) can be written as

$$\begin{array}{cc} \frac{\partial }{\partial w_{ij}\left(a,b\right)}[\sum_{n=1}^{N}(\sum_{i=1}^{L}v_{i}\left(x_{i}\right)+\sum_{i<j}^{L}w_{ij}\left(x_{i},x_{j}\right))-\text{log}Z] & =N_{ij}q\left(x_{i}=a,x_{j}=b\right)\\ & -N_{ij}\;p\left(x_{i}=a,x_{j}=b|v,w\right)\;, \end{array}$$ (31)

where $N_{ij}=\sum_{n=1}^{N}\omega_{n}I\left(x_{ni}\;\neq \;0,x_{nj}\;\neq \;0\right)$ is the summed weight of sequences that have no gap in either column *i* or *j*, $q\left(x_{i}=a,x_{j}=b\right)=\frac{1}{N_{ij}}\sum_{n=1}^{N}\omega_{n}I\left(x_{ni}=a,x_{nj}=b\right)$ represents the empirically observed pairwise amino acid frequencies that are normalized over *a*, *b* ∈ {1, …, 20}, and *p*(*x*_*i*_ = *a*, *x*_*j*_ = *b*|**v**, **w**) corresponds to the model probabilities of the MRF for observing an amino acid pair (*a*, *b*) at positions *i* and *j*. The empirical amino acid counts, given by *N*_*ij*_*q*(*x*_*i*_ = *a*, *x*_*j*_ = *b*), are constant and need to be computed only once from the alignment.

The marginal distributions of the MRF cannot be computed analytically as it involves the normalization constant *Z*. Markov chain Monte Carlo (MCMC) algorithms can be used to generate samples from probability distributions that involve the computation of complex integrals such as the normalization constant *Z*. Given that the Markov chains run long enough, the equilibrium statistics of the samples will be identical to the true probability distribution statistics. Thus, an estimate of the marginal distribution of the MRF in the gradient in Eq 31, *p*(*x*_*i*_ = *a*, *x*_*j*_ = *b*|**v**, **w**), can be obtained by simply computing the expected amino acid counts from MCMC samples. However, MCMC methods require many sampling steps to obtain unbiased estimates from the stationary distribution which comes at high computational costs.

Hinton suggested contrastive divergence (CD) as an approximation to MCMC methods [55]. The idea is simple: instead of starting a Markov chain from a random point and running it until it has reached the stationary distribution, we run *C* chains in parallel, each being initialized with one of the sequences from the input MSA and we evolve them for only a small number of steps. Obviously the chains do not converge to the stationary distribution in only a few steps and the sequence samples obtained from the current configuration of the chains present biased estimates. The intuition behind CD is that even though the resulting gradient estimate from the biased samples will also be noisy and biased, it points roughly into the same direction as the true gradient of the full likelihood. Therefore the approximate CD gradient should become zero approximately where the true gradient of the likelihood becomes zero.

We apply CD and generate sequence samples to estimate the marginal probabilities by evolving Markov chains which have been initialized with randomly selected protein sequences from the original Pfam MSAs for one full step of Gibbs sampling. We set the number of Markov chains to *C* = max(500, 0.1*N*), with *N* being the number of sequences in the MSA, which seems to give a good trade-off between performance and runtime. Gibbs sampling requires updating at each sampling step all sequence positions *x*_*i*_ with *i* ∈ {1, …, *L*} (*L* = sequence length). For each position, a new amino acid *a* is chosen according to the conditional probability

$$p\left(x_{i}^{t+1}\;=a|x_{-i}^{t},v,w\right)\propto \text{exp}(v_{i}\left(a\right)+\sum_{j\neq i}^{L}\;w_{ij}\left(a,x_{j}^{t}\right)).$$ (32)

This Gibbs sampling approach is known to generate samples **x**^0^, …, **x**^*t*^ that are distributed according to the model probability in Eq 11 [56, 57]. Note that we do not update positions representing a gap and we thereby retain the gap structure of the initial sequence.

A modification of CD known as persistent contrastive divergence (PCD) does not reinitialize the Markov chains at data samples every time a new gradient is computed [46]. Instead, the Markov chains are kept persistent: they are evolved between successive gradient computations. The assumption behind PCD is that the model changes only slowly between parameter updates given a sufficiently small learning rate. Consequently, the Markov chains will not be pushed too far from equilibrium after each update but rather stay close to the stationary distribution [46, 58, 59].

Tieleman and others observed that PCD performs better than CD in all practical cases tested, even though CD can be faster in the early stages of learning [46, 58, 60]. Therefore we start optimizing the full likelihood with CD and switch to PCD at later stages of learning. CCMpredPy settings for training a MRF with persistent contrastive divergence are listed in S1 Text.

### Generating MCMC samples from MRFs with CCMgen

MCMC samples for the analysis in Fig 1 have been generated with CCMgen by evolving 10000 Markov chains by repeated Gibbs sampling as described in Eq 32. The Markov chains, each representing protein sequences of length *L* (length of protein in the PSICOV data set) have been randomly initialized with the 20 amino acids. Since the alignment substructure is strongly impacted by the non-random distribution of gaps in the sequences (S2a Fig), in a second step the gap structure of randomly selected sequences from the original Pfam alignment is copied over (gaps represented as 21st amino acid). Thus, it is ensured that the sampling procedure reproduces the original alignment substructure as closely as possible (S2b and S2c Fig). The number of Gibbs steps before drawing samples was set to 500. Increasing the number of Gibbs steps to e.g. 1000 does not change the statistics of the MCMC samples, hence we can assume that the Markov chains have reached the equilibrium distribution. CCMgen settings for MCMC sampling are listed in S1 Text.

### Sampling sequences from MRFs along phylogenies with CCMgen

Instead of evolving sequences along a linear path, the MRF model can also be used to sample protein sequences according to an arbitrary phylogenetic tree.

CCMgen can simulate the evolution of sequences along any given phylogenetic tree constrained by a MRF model, such as those calculated from CCMpred for example. The user can either supply a phylogenetic tree in Newick format that has been generated by a phylogenetic reconstruction program such as FastTree [61] on a real alignment or choose between two types of idealized trees, a binary and a star-shaped topology. For these idealized trees the user can specify the number of leaf nodes and the total depth of the tree, which is the total number of mutations per position from the sequence at the root to the leaf nodes. The root sequence can either be supplied by the user or be generated by evolving an all-alanine sequence with a number of mutations (i.e. Gibbs sampling steps according to the MRF as described in Eq 32). Sequences at subsequent child nodes are generated one by one, by duplicating the sequence at the parent node and evolving the respective child node sequences each with a number of mutations proportional to the edge length. The output of CCMgen is a MSA file with the sequences at the leaf nodes of the tree. CCMgen is released as open-source python command-line application.

#### Workflow for quantification of noise in contact prediction with CCMgen

We used CCMpredPy to learn MRF models for all Pfam alignments in the PSICOV data set using PCD. In order to obtain models with few but precise constraints, we set coupling parameters to zero for non-contacting residue pairs (*C*_*β*_ distance > 12Å) by initializing them to zero and setting the gradients to zero in the optimization. This procedure ensures that the majority of residue pairs not forming contacts in the protein structure will not be coupled in the MRF model.

We used CCMgen with the learned MRF models to generate synthetic alignments by evolving sequences along idealized star and binary tree topologies. The ancestral sequence at the root of a tree, **x**^*t*^ with *t* = 0, is obtained by evolving an all-alanine sequence for 10 steps of Gibbs sampling as described in Eq 32. The synthetic alignments have the same number of sequences as the corresponding Pfam alignments from the PSICOV set.

We also ensure that the diversity of the resulting MSAs is within 1% of the diversity of the original Pfam MSA from the PSICOV set by adjusting the depth of the trees, which is equivalent to adapting the mutation rate (S5a Fig). We measure diversity as the number of effective sequences, *N*_eff_, defined as the exponential of the average column entropies, as defined in the HH-suite software package [62].

For each new CCMgen run with adapted mutation rate a new ancestral sequence is sampled by evolving an all-alanine sequence for 10 Gibbs steps. We found that the aforementioned procedure of masking non-contacting residue pairs during training of the MRF model has the advantageous effect of allowing smaller mutation rates to be used to achieve the desired diversity compared to sampling sequences with a fully parametrized model, likely due to the smaller number of constraints trapping the sampling procedure in local optima. Enforcing small mutation rates is essential for preserving the interdependence between sequences when sampling along binary topologies, which consequently controls the amount of phylogenetic bias. The mutation rates used to obtain synthetic alignments of similar diversity as the original Pfam alignments are very similar regardless of the phylogenetic topology along which sequences were sampled (S5b Fig).

CCMpredPy and CCMgen settings for training the MRF with persistent contrastive divergence and generating the synthetic alignments along binary and star tree topologies are listed in S1 Text.

### Dataset and preprocessing

We used the PSICOV data set that was published together with the PSICOV method [49] and which comprises MSAs for 150 Pfam domains with known crystal structures. For each Pfam MSA in the PSICOV set we first removed sequences with more than 75% gaps and columns with more than 50% gaps, similarly as in [50, 51, 63], to reduce the well-known impact of gaps on the analysis.

## Supporting information

S1 Text Running CCMpredPy and CCMgen. Parameter settings used with CCMpredPy and CCMgen in this study.
(PDF)

S2 Text Analysing alignment substructure with principal component analysis (PCA). (PDF)

S1 Fig Comparing quality of Markov random field (MRF) models learned with pseudo-likelihood maximization and persistent contrastive divergence (PCD). (PDF)

S2 Fig Visualizing alignment substructure for protein 1gmxA by projecting sequences of the multiple sequence alignment (MSA) onto their first two principal components. (PDF)

S3 Fig Contact scores computed from Markov random field (MRF) models trained with pseudo-likelihood maximization and persistent contrastive divergence (PCD) correlate strongly. (PDF)

S4 Fig Distribution of Pearson correlation coefficients between average product correction (APC) and entropy bias correction (EntC) term. (PDF)

S5 Fig Statistics of synthetic alignments generated with CCMgen along binary and star tree topologies. (PDF)
