# Supplementary material for: Synthetic protein alignments by CCMgen quantify noise in residue-residue contact prediction
Source: PLoS Comput Biol. 2018 Nov 5;14(11):e1006526. doi: 10.1371/journal.pcbi.1006526 (PMC6237422; doi:10.1371/journal.pcbi.1006526)
Supplement: S1 Text — Parameter settings used with CCMpredPy and CCMgen in this study. (PDF) [file pcbi.1006526.s001.pdf]

S1 TEXT. RUNNING CCMPREDPY AND CCMGEN

CCMpredPy was run with the following settings for training Markov random fields (MRFs) with pseudo-likelihood maximization:

```
--ofn-pll --maxit 5000 --max-gap-seq 75 --max-gap-pos 50
```

CCMpredPy was run with the following settings for training MRFs with persistent contrastive diverence:

```
--ofn-cd --persistent --maxit 5000 --max-gap-seq 75 --max-gap-pos 50
```

CCMgen was run with the following settings to generate Markov chain Monte Carlo (MCMC) samples:

```
--max-gap-pos 50 --max-gap-seq 75 --mcmc-sampling --mcmc-sample-random-gapped
--mcmc-burn-in 500 --num-sequences 10000
```

CCMgen was run with the following settings to generate sequence samples along a star or binary tree topology:

```
--max-gap-pos 50 --max-gap-seq 75 --tree-[star|binary] --mutation-rate-neff
--burn-in 10
```
