# Supplementary material for: Synthetic protein alignments by CCMgen quantify noise in residue-residue contact prediction
Source: PLoS Comput Biol. 2018 Nov 5;14(11):e1006526. doi: 10.1371/journal.pcbi.1006526 (PMC6237422; doi:10.1371/journal.pcbi.1006526)
Supplement: S2 Text — (PDF) [file pcbi.1006526.s002.pdf]

S2 TEXT. ANALYSING ALIGNMENT SUBSTRUCTURE WITH PRINCIPAL COMPONENT ANALYSIS (PCA)

A multiple sequence alignment (MSA) with N sequences $x^n = (x_i, ..., x_L)$ of length L can be represented as a data maxtrix of L categorical variables with 21 states (20 amino acids and gap symbol). In order to visualize the substructure of alignments we applied a principal component analysis (PCA) analysis on the MSA. The protein sequences of the MSA were converted to binary vectors using one-hot encoding and PCA was then performed on the transformed data matrix. Sequences were projected onto the first two principal components.
