# Supplementary material for: Synthetic protein alignments by CCMgen quantify noise in residue-residue contact prediction
Source: PLoS Comput Biol. 2018 Nov 5;14(11):e1006526. doi: 10.1371/journal.pcbi.1006526 (PMC6237422; doi:10.1371/journal.pcbi.1006526)
Supplement: S1 Fig — (PDF) [file pcbi.1006526.s003.pdf]

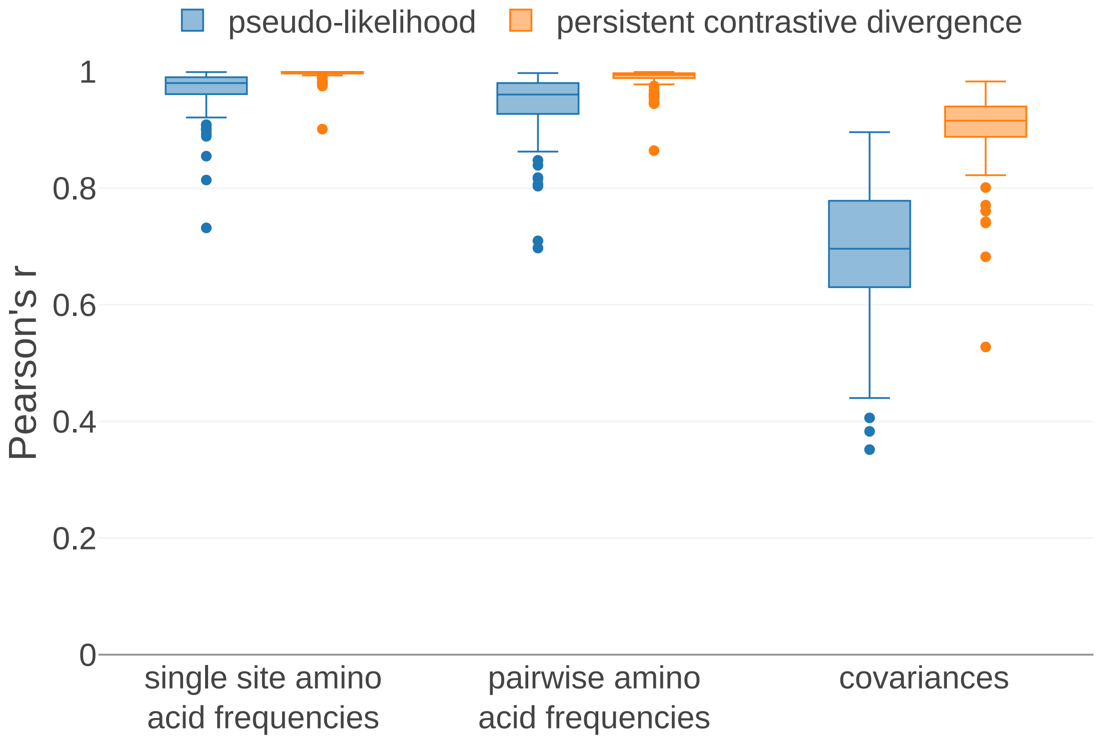

S1 Fig. Comparing quality of Markov random field (MRF) models learned with pseudo-likelihood maximization and persistent contrastive divergence (PCD). The Pearson correlation of alignment statistics (single-site and pairwise amino acid frequencies and covariances) between original Pfam alignments and MCMC samples obtained from the MRF models serves as proxy to evaluate model quality. Distribution of Pearson correlation coefficients is shown for all 150 proteins in the PSICOV dataset.
