# Supplementary material for: Synthetic protein alignments by CCMgen quantify noise in residue-residue contact prediction
Source: PLoS Comput Biol. 2018 Nov 5;14(11):e1006526. doi: 10.1371/journal.pcbi.1006526 (PMC6237422; doi:10.1371/journal.pcbi.1006526)
Supplement: S2 Fig — (PDF) [file pcbi.1006526.s004.pdf]

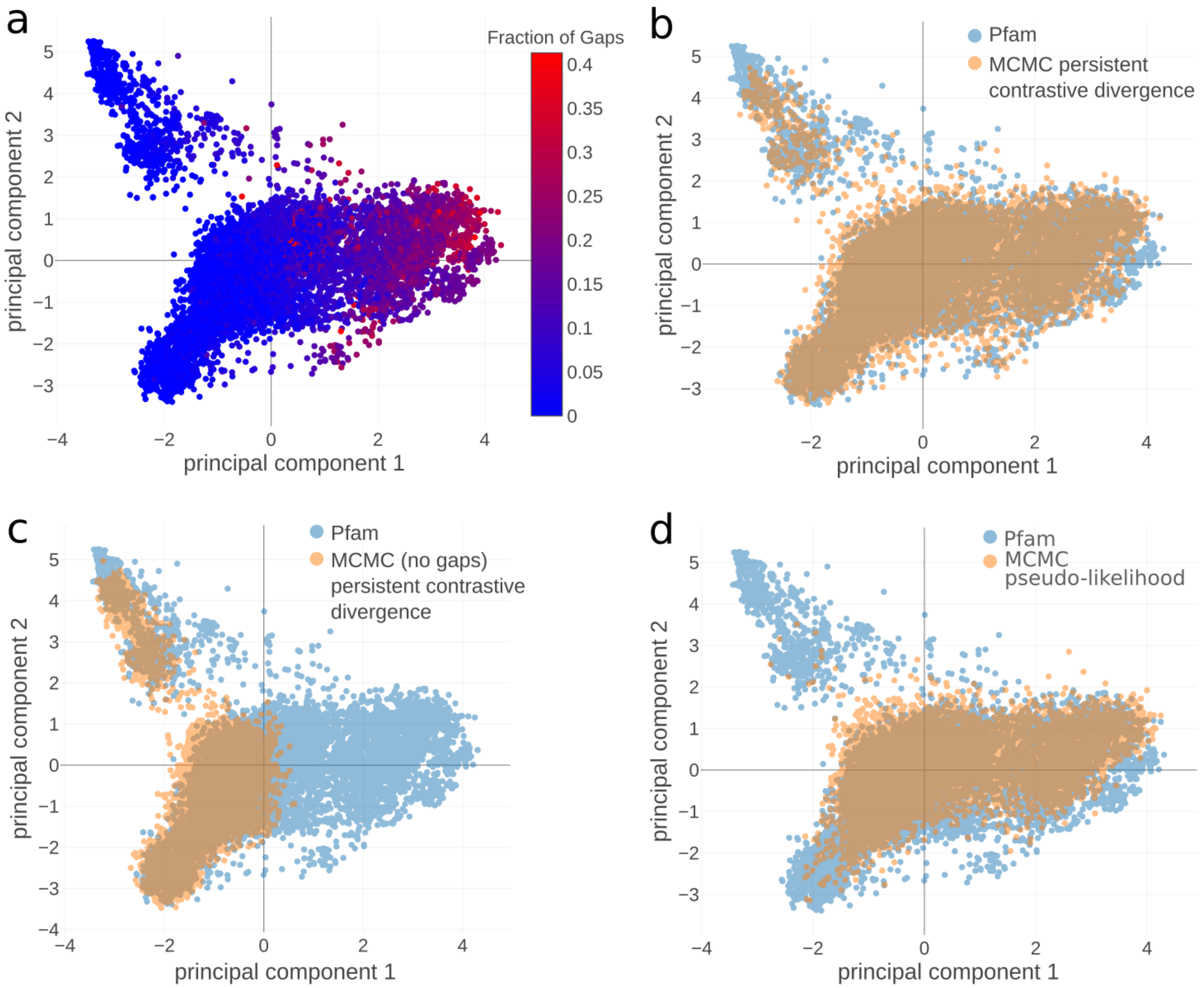

S2 Fig. Visualizing alignment substructure for protein 1gmxA by projecting sequences of the multiple sequence alignment (MSA) onto their first two principal components. (a) Original Pfam alignment. The color gradient encodes the fraction of gaps within each sequence of the multiple sequence alignment (MSA). Clusters in the projection plot might represent protein family substructure as well as similar distribution of stretches of gaps. **(b)** Sequences generated via Markov chain Monte Carlo (MCMC) sampling from the Markov random field (MRF) model trained with persistent contrastive divergence (PCD). The Markov chains for MCMC sampling have been initialized as random sequences comprised of the 20 amino acids and subsequently the gap structure of randomly selected sequences from the Pfam alignment has been copied over. Sequence positions representing gaps are not updated during the sampling proceess, so that the gap structure of the Pfam alignment is retained. **(c)** Sequences generated via MCMC sampling from the MRF model trained with PCD. The Markov chains for MCMC sampling have been initialized as random sequences of the 20 amino acids with no additional gap state. **(d)** Same as in **(b)** but using the MRF model trained with pseudo-likelihood for MCMC sampling. The sequence space represented by sequences of the original Pfam alignment in the upper left corner and lower left corner is not covered by sequences from the MCMC sample.
