# Supplementary material for: Synthetic protein alignments by CCMgen quantify noise in residue-residue contact prediction
Source: PLoS Comput Biol. 2018 Nov 5;14(11):e1006526. doi: 10.1371/journal.pcbi.1006526 (PMC6237422; doi:10.1371/journal.pcbi.1006526)
Supplement: S3 Fig — (PDF) [file pcbi.1006526.s005.pdf]

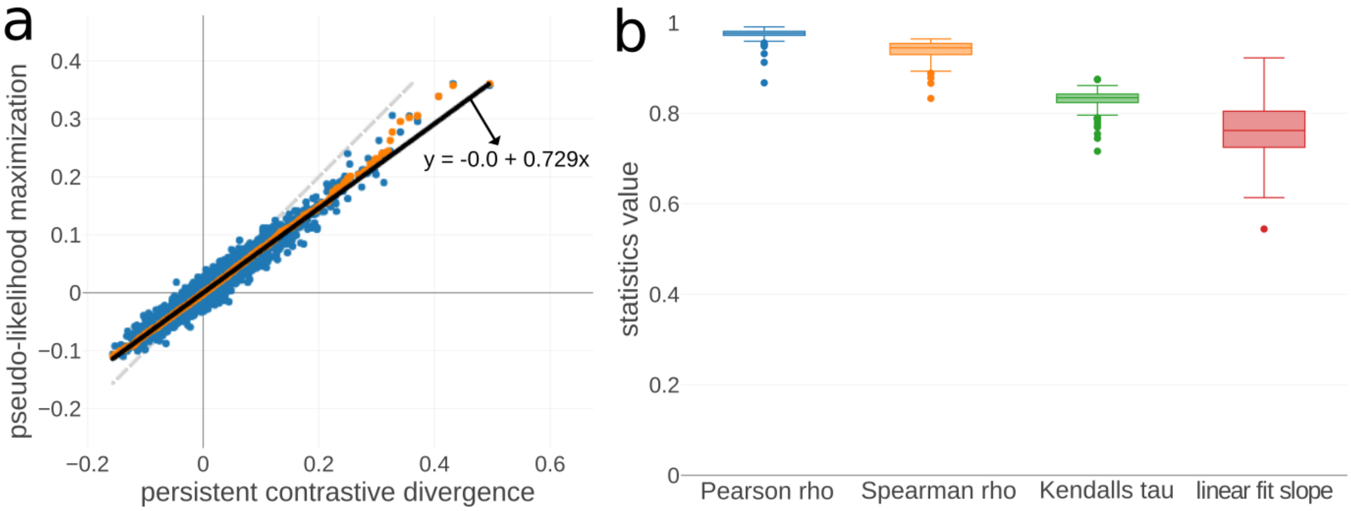

S3 Fig. Contact scores computed from Markov random field (MRF) models trained with pseudo-likelihood maximization and persistent contrastive divergence (PCD) correlate strongly. Contact scores are computed as average product correction (APC) corrected L_2-norm of coupling coefficients from MRFs models that have been learned either with pseudo-likelihood maximization or PCD. **(a)** Contact scores for protein 1bkrA in the PSICOV dataset (shown in blue) are highly correlated (Pearson r=0.97) and tend to be stronger when obtained with PCD than compared to pseudo-likelihood inference (using identical regularization settings): a linear regression fit (regression line shown in black) yields a slope = 0.73. For contact prediction the exact value of contact scores is not of importance but the correct ranking of residue pairs. The ranking of residue pairs is also almost identical (Spearman rho = 0.96, Kendall tau = 0.84). A quantile-quantile plot of the sorted contact scores (shown in orange) confirms that the pseudo-likelihood scores are more dispersed than the PCD scores. **(b)** Distribution of correlation statistics between contact scores for all proteins in the PSICOV dataset. The correlation as well as rank correlation between contact scores of both methods is strong. The slopes of linear regression fits for contact scores between both methods (as shown in **(a)**) is always positive but smaller than 1, indicating that contact scores computed from the pseudo-likelihood model are generally smaller than contact scores computed from the PCD model.
