# Supplementary material for: Synthetic protein alignments by CCMgen quantify noise in residue-residue contact prediction
Source: PLoS Comput Biol. 2018 Nov 5;14(11):e1006526. doi: 10.1371/journal.pcbi.1006526 (PMC6237422; doi:10.1371/journal.pcbi.1006526)
Supplement: S4 Fig — (PDF) [file pcbi.1006526.s006.pdf]

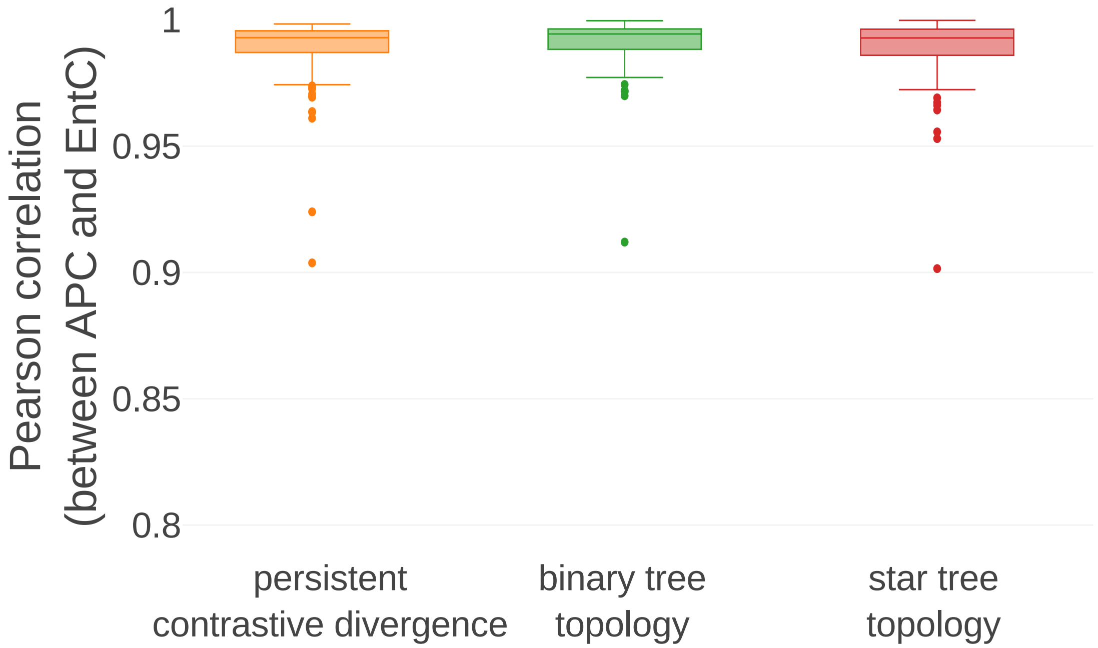

S4 Fig. Distribution of Pearson correlation coefficients between average product correction (APC) and entropy correction (EntC) term. The left boxplot refers to Markov random field (MRF) models learned from the Pfam alignments in the PSICOV dataset with persistent contrastive divergence (PCD). The boxplot in the center and on the right relates to MRF models that have been learned with PCD from synthetic alignments generated with CCMgen as described in Material and Methods along binary tree and star tree topologies, respectively. In order to compute the APC term, the contact maps have been computed with L_2-norm score of the coupling coefficients from MRF models.
