# Supplementary material for: Synthetic protein alignments by CCMgen quantify noise in residue-residue contact prediction
Source: PLoS Comput Biol. 2018 Nov 5;14(11):e1006526. doi: 10.1371/journal.pcbi.1006526 (PMC6237422; doi:10.1371/journal.pcbi.1006526)
Supplement: S5 Fig — (PDF) [file pcbi.1006526.s007.pdf]

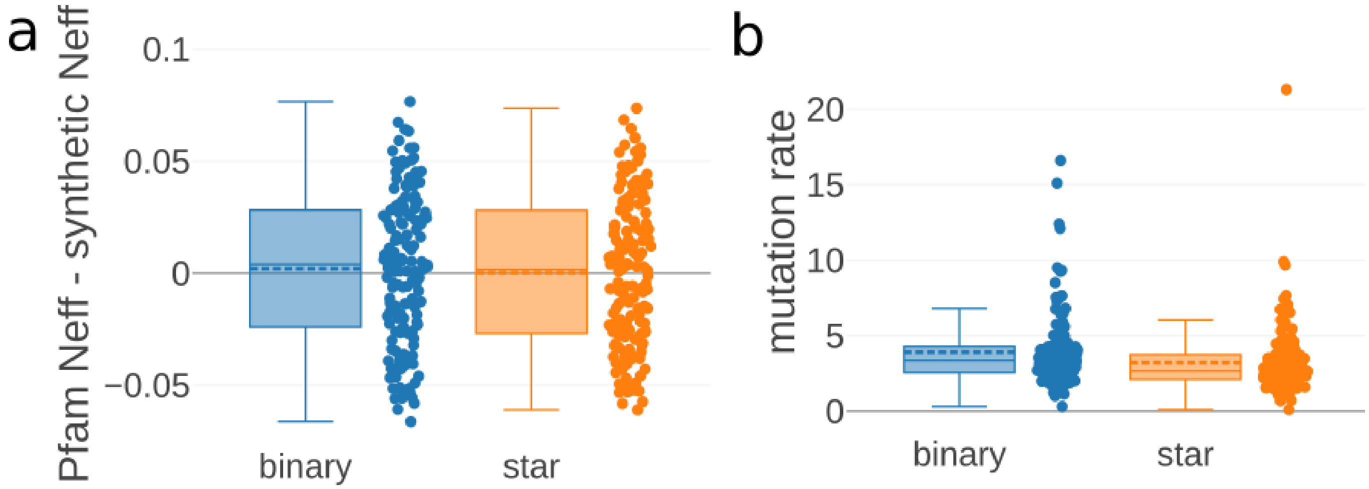


**S5 Fig. Statistics of synthetic alignments generated with CCMgen along binary and star tree topologies.** Markov random field (MRF) models have been trained with PCD for the 150 Pfam alignments in the PSICOV dataset. Synthetic alignments comprise same number of sequences as original Pfam alignments and have been generated such that their diversity is comparable to the diversity of the original Pfam alignment (as described in Material and Methods). **(a)** Difference between the diversity (number of effective sequences, Neff as described in Material and Methods) of the original Pfam alignment and the generated synthetic alignments. **(b)** Mutation rate used to generate synthetic alignments of comparable diversity as orginal Pfam alignments. Dashed line in boxplots represents the mean, solid line represents the median of the distribution.
